# First Report of Fecal Microflora of Wild Bar-Headed Goose in Tibet Plateau

**DOI:** 10.3389/fvets.2021.791461

**Published:** 2022-01-10

**Authors:** Shixiong Dong, Shijun Xu, Jian Zhang, Riaz Hussain, Hong Lu, Yourong Ye, Khalid Mehmood, Hui Zhang, Peng Shang

**Affiliations:** ^1^College of Animal Science, Tibet Agriculture and Animal Husbandry College, Linzhi, China; ^2^The Provincial and Ministerial Co-founded Collaborative Innovation Center for R & D, Tibet Agricultural and Animal Husbandry Resources, Linzhi, China; ^3^Faculty of Veterinary and Animal Sciences, Islamia University of Bahawalpur, Bahawalpur, Pakistan; ^4^College of Veterinary Medicine, South China Agricultural University, Guangzhou, China

**Keywords:** wild, bar-headed goose, 16SrDNA, fecal microflora, Tibet plateau

## Abstract

The bar-headed goose (*Anser indicus*) has two black spots on its head. It is considered an important bird in China. It breeds in plateau lakes, especially saltwater lakes, and swamp areas. However, the intestinal flora of wild bar-headed geese in the Tibet Autonomous Region is currently not known. In this study, 16S rDNA sequencing was performed on the intestinal microbes of wild bar-headed geese. A total of 513,505 reads of raw data were obtained, and the results analyzed the average number of 128,376 ± 2,392 reads per sample. The microbiota of all samples consists of 10 main bacterial phyla, including Firmicutes, Proteobacteria, Bacteroidetes, Actinobacteria, Cyanobacteria, Patescibacteria, Deferribacteres, Planctomy-cetes, Fusobacteria, and Tenericutes. The results indicated that Firmicutes (67.34%) was the predominant phylum, followed by Proteobacteria (29.03%) and Cyanobacteria (1.97%). In our research, we identified the intestinal flora of the wild bar-headed goose, which provides valuable information for further research on the gene function of the bar-headed goose and the intestinal flora of wild animals. These findings are also useful and valuable for genetic and high-altitude research in the Tibet Autonomous Region.

## Introduction

Research on the development and progress of biotechnology (1) has shown that gut microbes not only affect the health of the host but also play an important role in the occurrence of diseases including obesity (2), cancer (3), diabetes (4), and increased cardiovascular risks through different metabolic processes (5). Among vertebrates, birds are considered to be the most common and diverse on earth, with more than 10,000 living species (6). It is reported that a variety of birds in the world constantly migrate from one place to another every year. They can migrate over vast geographical areas and cross biological and geographical boundaries (7). Birds can act as a source of infections for public health and animals through direct contact or as carriers of pathogens including avian and zoonotic pathogens and antibiotic-resistant bacteria (8). For these reasons, birds play an important role in the spread of microorganisms from one place to another, thereby affecting their dynamics ecology and the evolution of various viruses and bacteria. In the published literature, research on the gut microbiota of birds is very limited and most of them are researches on some artificially farmed economic species.

The bar-headed geese are a species unique to Asia, and most bar-headed geese breed in different places during the winter season including Tibet, south central Tibet, and India (9, 10). During migration, the bar-headed geese avoid areas with high altitudes, harsh climates, and barren vegetation and find suitable habitats that are convenient for harvesting food. Birds may carry and spread different infectious agents during long-distance migration including avian influenza virus and other pathogens that pose a threat to wildlife and public health.

In recent years, advancements in sequencing technology have rapidly improved (researchers' ability in) understanding of the intestinal microbiota of different animals including humans, mice, and other mammals. However, the application of sequencing technology in the study of bird intestinal flora is still limited (11). Compared with mammals, the gastrointestinal tract of birds is relatively short, and the time to digest food is also short, which enables them to form highly selective and adaptable microbiota. Studies have shown that the diversity of an intestinal bacterial community of white-headed cranes in different regions has been investigated using 16S rDNA sequencing techniques. Previous studies have found that there are significant differences in the structure and diversity of the intestinal bacterial community of hooded cranes in different locations in winter and different climatic conditions that affect the composition of the intestinal bacterial community (12). Few studies have reported sequencing techniques for feeding habits (insectivorous or omnivorous) to determine the differences in the digestive tract microbiota of passerine birds like those in New Guinea (13). At present, there are few reports on the use of 16S rDNA sequencing technology to study the intestinal microbes of the wild bar-headed geese in Tibet. Therefore, this study used 16S rDNA sequencing technology to analyze the intestinal microbial diversity of wild bar-headed geese in Tibet.

## Materials and Methods

### Sample Collection

**Figure 1:**
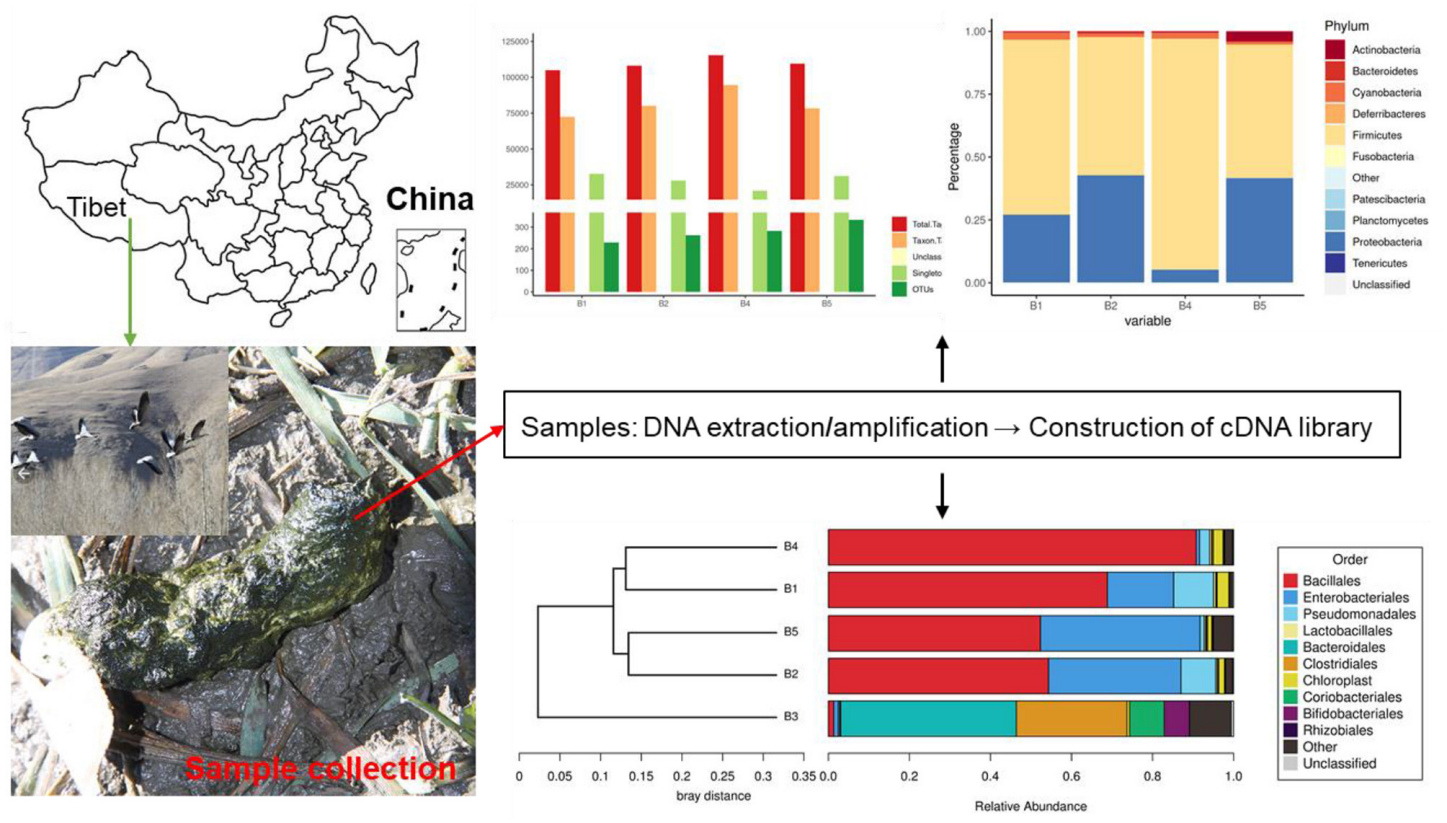
Research method and geographic location of this study.

Fecal samples were collected from four different bar-headed geese living in the southern part of Naidong District, Shannan City (29°10′46″N, 91°46′15″E, elevation 3,552 m), China. Fresh fecal samples were collected and stored in sterile test tubes. All the freshly excreted fecal samples by bar-headed geese were collected, and the middle part of the sample was processed (Figure 1). All samples were transported to the laboratory using a −20°C portable refrigerator and stored at −80°C until further processing.

### DNA Extraction, PCR Amplification, and High-Throughput Sequencing

Microbial DNA was extracted using the HiPure Stool DNA Kits (Magen, Guangzhou, China) according to the manufacturer's guidelines. The extracted DNA was quantified and evaluated for purity using NanoDrop 2000 UV-vis spectrophotometer and 1% agarose gel electrophoresis. The hypervariable region V3–V4 of the 16S rDNA genes was amplified using primers 341F(5′-CCTACGGGNGGCWGCAG-3′) and 806R(5′-GGACTACHVGGGTATCTAAT-3′) (14). The PCR conditions were as follows: initial denaturation at 94°C for 2 min, denaturation at 98°C for 10 s, 62°C annealing for 30 s, 68°C extension for 30 s using 30 cycles, and a final extension at 68°C for 5 min. PCR reactions were performed in triplicate 50 μl mixture containing 5 μl of 10 × KOD buffer, 5 μl of 2 mM dNTPs, 3 μl of 25 mM MgSO_4_, 1.5 μl of each primer (10 μM), 1 μl of KOD polymerase, and 100 ng of template DNA. Amplicons were extracted on 2% agarose gel and purified using the AxyPrep DNA Gel Extraction Kit (Axygen Biosciences, Union City, CA, USA) according to the manufacturer's instructions. All the amplicons were quantified using ABI Step One Plus Real-Time PCR System (Life Technologies, Foster City, USA). The purified amplicons were pooled in equimolar and paired-end sequenced (2 × 250) on an Illumina platform according to the standard protocols.

### Bioinformatic and Statistical Analyses

Raw data containing adapters or low quality reads may affect the following assembly and analysis. In order to get high quality clean reads, the raw reads were further filtered following the guidelines using FASTP to remove the reads containing more than 10% of unknown nucleotides and to remove the reads containing less than 80% of bases with quality (*Q*-value) > 20. After that the paired end clean reads were merged as raw tags using FLSAH (15) with a minimum overlap of 10 bp and mismatch error rates of 2%. Noisy sequences of raw tags were filtered by QIIME (16) pipeline under specific filtering conditions (17) to obtain the high-quality clean tags. Clean tags were searched against the reference database (http://drive5.com/uchime/uchime_download.html) to perform reference-based chimera checking using UCHIME algorithm (http://www.drive5.com/usearch/manual/uchime_algo.html). After that, all the chimeric tags were removed, and effective tags were finally obtained and used for further analysis. The effective tags were clustered into operational taxonomic units (OTUs) of ≥ 97% using UPARSE (18) pipeline. The tag sequence with highest abundance was selected as representative sequence within each cluster. The representative sequences were classified into organisms by a naive Bayesian model using RDP classifier (19) based on SILVA (20) Database (https://www.arb-silva.de/), with the confidence threshold values ranging from 0.8 to 1.

The abundance statistics of each taxonomy were visualized using Krona (21). The stacked bar plot of the community composition was visualized in R project ggplot2 package (version 2.2.1). Chao1, Simpson, and all other alpha diversity indexes were calculated in QIIME.FAPROTAX database (Functional Annotation of Prokaryotic Taxa), and associated software (22) (version 1.0) were used for generating the ecological functional profiles of bacteria. The Kyoto Encyclopedia of Genes and Genomes (KEGG) pathway analysis of the OTUs was inferred using Tax4Fun (version 1.0) or PICRUSt (version 2.1.4).

### Statistical Analysis

The abundance statistics of each taxonomy were visualized using Krona (21). Chao1, Simpson and all other alpha diversity indexes were calculated in QIIME. Alpha index comparison between groups was calculated by Welch's *t*-test and Wilcoxon rank test in R project. FAPROTAX database and associated software (22) (version 1.0) were used for generating the ecological functional profiles of bacteria. Analysis of function difference between groups was calculated by Welch's *t*-test, Wilcoxon rank test, Kruskal–Wallis *H*-test, and Tukey's honestly significant difference (HSD) test in R Project Vegan package (version 2.5.3).

## Results

### Effective Sequence Quality Assessment

Results on the microbiome analysis indicated that totals of 118,238, 120,100, 123,894, and 121,612 original sequences were acquired from B1, B2, B3, and B4 groups, respectively (Table 1). After eliminating the unqualified data, a total number of 474,918 high-quality tags were achieved from all the samples. The length distribution of all samples ranged from 200 to 475 bp.

**Table 1:** Tags and OTUs quantity statistics.

**Sample ID**	**Raw tags**	**Clean tags**	**Chimera**	**Effective tags**	**Effective ratio (%)**	**OTUs**
B1	118,238	115,881	10,860	105,021	83.38	229
B2	120,100	118,191	10,127	108,064	85.04	262
B3	123,894	121,422	6,020	115,402	87.83	282
B4	121,612	119,424	9,869	109,555	84.88	333

**Figure 2:**
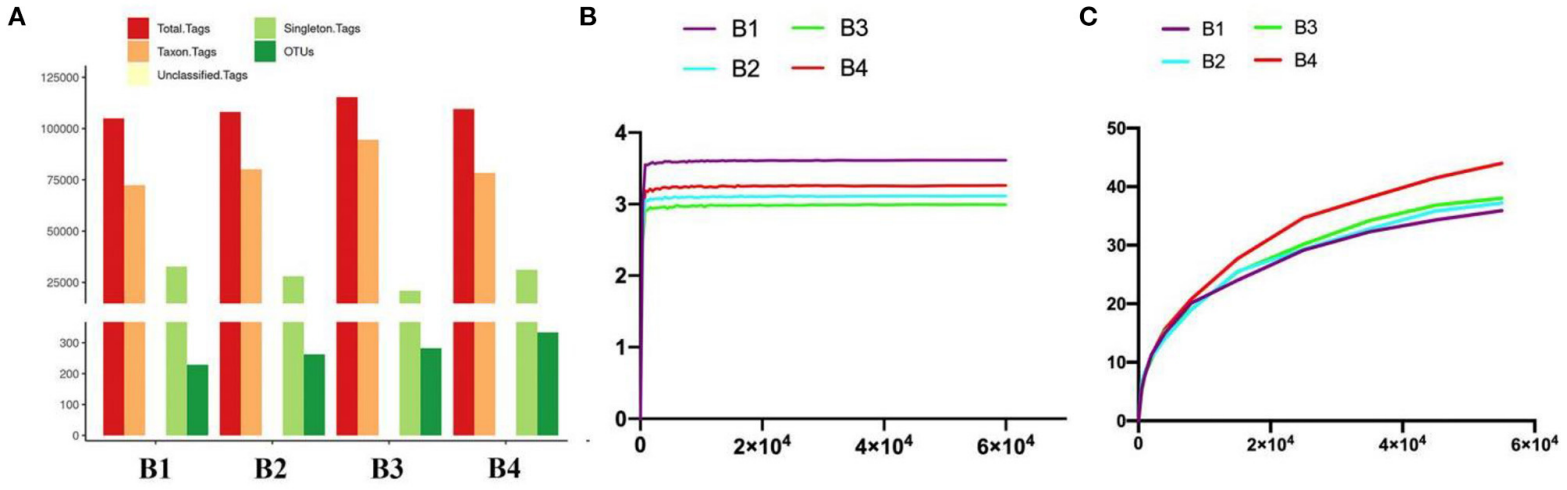
Gut bacterial OTU distribution and feasibility analysis. **(A)** OTUs information of four bar-headed goose samples (B1–B4). **(B)** Shannon diversity index curve. **(C)** PD diversity index curve.

All the optimized sequences are aligned to OTU representative sequences by UPARSE software, and sequences with more than 97% similarity with representative sequences are selected to generate OTUs. After classification matching, a total of 1,106 OTUs (B1 = 229, B2 = 262, B3 = 282, B4 = 333) were obtained (Figure 2A).

### The Diversity of the Intestinal Microbial

Generally, the alpha diversity of the gut microbial community can be analyzed by mainstream alpha diversity indexes including species (Sob), Chao1, ACE, Shannon, Simpson, Good's Coverage, pielou, and PD-whole tree. Our results showed that both the Shannon curve and the PD-whole tree curve have reached the plateau, indicating that the sequencing results can reflect the diversity of the present samples. Furthermore, the rank abundance curve is wide and the downward trend is flat, showing excellent abundance and evenness (Figures 2B,C and Table 2). The repetition between samples is well, and it can effectively reflect the bar-headed geese that live in Tibet.

**Table 2:** Alpha diversity index.

**Index**	**Shannon**	**Simpson**	**Chao**	**Ace**
B1	3.615065736	0.880361454	261.8095238	270.8105696
B2	3.11525356	0.802646058	317.122449	336.3319194
B3	2.993777108	0.797401177	293.9583333	307.5159008
B4	3.261948332	0.793011583	367.7560976	392.4399574

### Microbial Community Structure Analysis

**Figure 3:**
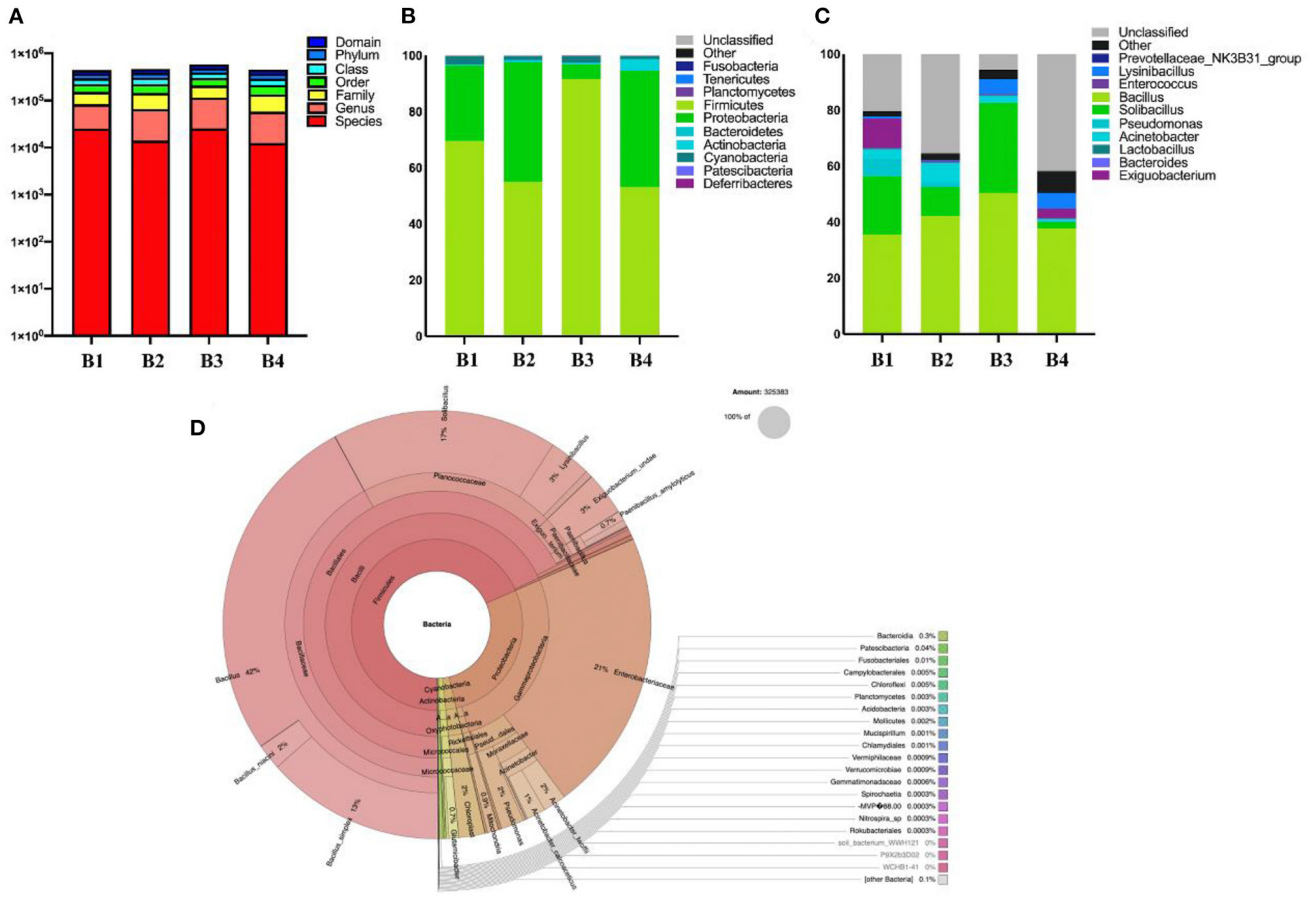
The gut bacterial diversities in wild bar-headed goose. **(A)** The sequence of each sample at each classification level constitutes a histogram (numerical value). **(B)** The classification of phylum intestinal microbiome classification composition. **(C)** Genus composition of intestinal microorganisms in the classification of genera. **(D)** Krona diagram of intestinal microorganisms.

This study analyzed the composition of intestinal microbes at the phylum level. The results on 16S rDNA gene analysis indicated the presence of high proportions of Firmicutes, Proteobacteria, and Cyanobacteria (Figure 3). As for the fecal samples of four groups (B1, B2, B3, and B4) of bar-headed geese, the main bacterial phylum was Firmicutes (69.60, 54.92, 91.76, and 53.09%), Proteobacteria (26.89, 42.64, 5.12, and 41.47%), and Cyanobacteria (2.94, 1.46, 2.43, and 1.03%), accounting for approximately 97% of the taxonomic groups identified (Figure 3A). The results indicated that the predominant phylum of the B3 group was Firmicutes (91.76%), and Proteobacteria (5.12%) was the secondary phylum (5.12%), which was significantly different from other groups. At the genus level, *Bacillus* (41.5%) was the most dominant genus, followed by *Solibacillus* (16.35%), *Exiguobacterium* (3.6%), *Acinetobacter* (3.34%), *Lysinibacillus* (3%), and *Pseudomonas* (2.1%) (Figures 3B–D).

### Function Prediction

**Figure 4:**
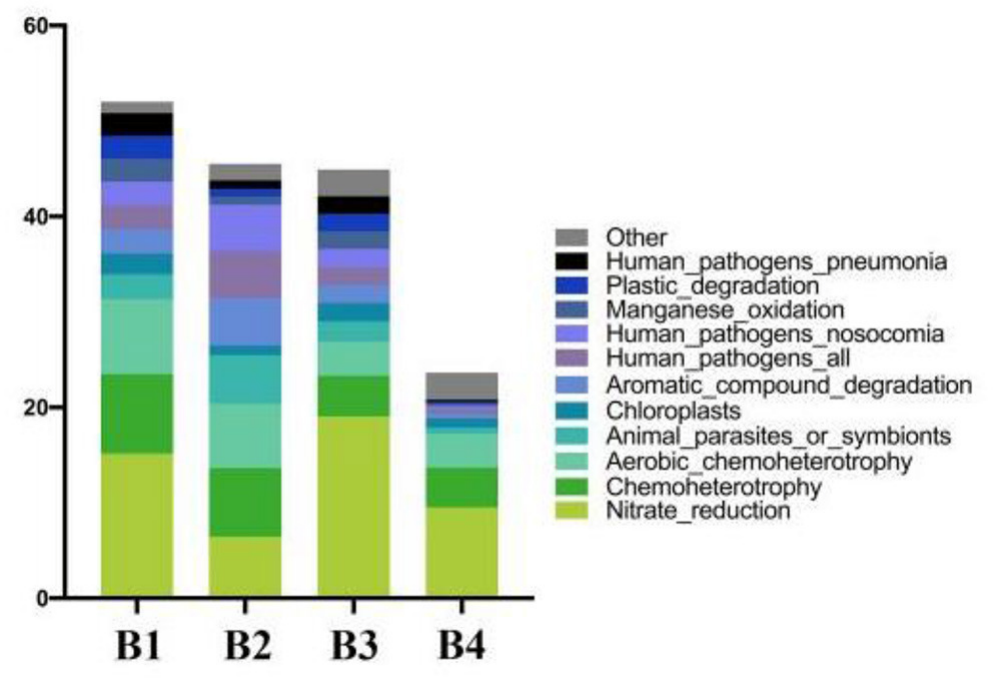
Prediction of intestinal microbial ecological function.

The horizontal coordinates of the stack diagram in samples are different, and the columns with different colors represent the relative abundance of different ecological functions. Among them, the results showed that the abundance of the top 11 functions and the abundance of other functions were combined into other categories (Figure 4).

Pathway results showed that bacteria were mainly related to metabolism, environmental information processing, genetic information processing, cellular processes, multiple levels of human diseases, and organismal systems (Table 3).

**Table 3:** Pathway classification table of four samples.

**Level_1**	**Level_2**	**B1**	**B2**	**B3**	**B4**
Metabolism	Carbohydrate metabolism	0.138262384	0.137204578	0.137924874	0.138646854
Metabolism	Amino acid metabolism	0.111421453	0.110412451	0.115661547	0.10842906
Metabolism	Metabolism of cofactors and vitamins	0.067729589	0.067289806	0.067904085	0.067051215
Metabolism	Energy metabolism	0.063731509	0.065070183	0.062474023	0.064980109
Metabolism	Nucleotide metabolism	0.055763617	0.054445485	0.056878619	0.054385279
Metabolism	Lipid metabolism	0.036512069	0.035869258	0.037546928	0.035530787
Metabolism	Xenobiotics biodegradation and metabolism	0.033547667	0.034390754	0.036494901	0.033232133
Metabolism	Glycan biosynthesis and metabolism	0.027226921	0.026742097	0.025474962	0.027337288
Metabolism	Metabolism of other amino acids	0.025496185	0.025942931	0.024702099	0.026259261
Metabolism	Metabolism of terpenoids and polyketides	0.027211244	0.026400639	0.030414398	0.025667158
Metabolism	Biosynthesis of other secondary metabolites	0.008173524	0.007898183	0.009028766	0.007621397
Environmental information processing	Membrane transport	0.129600582	0.133515969	0.130246536	0.134873702
Environmental information processing	Signal transduction	0.072893289	0.074424789	0.067582151	0.074795632
Environmental information processing	Signaling molecules and interaction	1.23E-05	1.38E-05	7.20E-06	1.53E-05
Genetic information processing	Translation	0.048245175	0.046507035	0.049168457	0.046347026
Genetic information processing	Replication and repair	0.045369871	0.04371493	0.046423147	0.043471616
Genetic information processing	Folding, sorting and degradation	0.024555915	0.023823158	0.024691151	0.023791536
Genetic information processing	Transcription	0.002134176	0.002049707	0.0021962	0.002040752
Cellular processes	Cell motility	0.020442414	0.022476729	0.018225513	0.023087706
Cellular processes	Cell growth and death	0.014260462	0.015056888	0.014708045	0.014753529
Cellular processes	Transport and catabolism	0.002376733	0.002398455	0.002395334	0.002351737
Cellular processes	Cell communication	1.02E-05	1.13E-05	4.88E-06	1.31E-05
Human diseases	Infectious diseases	0.032063795	0.031348205	0.027285025	0.032270163
Human diseases	Neurodegenerative diseases	0.002125349	0.002058065	0.002065167	0.002062671
Human diseases	Cancers	0.001735868	0.00184366	0.001730029	0.001826213
Human diseases	Endocrine and metabolic diseases	0.00062061	0.000628998	0.000629375	0.000635622
Human diseases	Immune diseases	0.000437665	0.000419475	0.000372922	0.000442945
Human diseases	Substance dependence	0.000168138	0.000198915	0.000177343	0.000189002
Human diseases	Cardiovascular diseases	5.48E-05	4.69E-05	6.73E-05	3.73E-05
Organismal systems	Endocrine system	0.002908448	0.002923953	0.002877012	0.002916835
Organismal systems	Environmental adaptation	0.002238247	0.002301476	0.001968817	0.002361541
Organismal systems	Digestive system	0.000908229	0.00084178	0.000837674	0.000848291
Organismal systems	Nervous system	0.000924544	0.000964138	0.000946395	0.000958608
Organismal systems	Immune system	0.000636844	0.000564746	0.000689687	0.000574406
Organismal systems	Excretory system	0.00013829	0.000135405	0.000131847	0.000136528
Organismal systems	Circulatory system	6.19E-05	6.51E-05	6.77E-05	5.78E-05
Organismal systems	Sensory system	9.89E-11	1.72E-08	1.72E-10	3.58E-10

## Discussion

Bar-headed geese not only survive on the plains but can also adjust to the hostile environment of the plateau. As an adaptive species of plateaus and plains, the bar-headed goose is one of the birds that efficiently pay attention (9), and some researchers have conducted special studies on the changes in oxygen in their bodies. Some researchers have also studied the temperature regulation (17) and childbirth (23) of bar-headed geese during their high-altitude migration. In this study, the 16S rDNA amplicons were sequenced for the first time in the feces of bar-headed geese in Tibet, and the diversity of intestinal microbes has also been investigated. Comparing this with previous studies on the microbial composition of birds at the phylum level, Firmicutes, Proteobacteria, Bacteroidetes, Actinobacteria, and Fusobacteria were dominant (24). The findings of this study regarding 16s rRNA sequencing are similar to an earlier study on the intestinal microbial diversity of cultured bar-headed goose (25). Previously, different studies also sequenced the diversity of the gut microbiota of bar-headed geese (wild, semi-captive, and captive), and the results indicated that the dominant phyla included Firmicutes, Proteobacteria, Actinobacteria, Bacteroidetes, and Fusobacteria (26) and in this study 23.33% *Turicibacter* in Firmicutes in the semi-artificial feeding group. A total of 77.67% *Lactococcus* in Firmicutes in the wild feeding group and 51% SMB53 in the feeding group were recorded. However, at the genus level in this study, *Bacillus* (41.5%) is the most dominant genus. Among Firmicutes (67.34%), Proteobacteria (29.03%) and Cyanobacteria (1.97%), the most dominant phylum is Firmicutes (67.34%). This may be due to the different living environment of the bar-headed geese. The samples in this study were collected from bar-headed geese living in Tibet more than 3,000 m above sea level where the growth of plants varies at different altitudes. Therefore, the variations in the results of our study from different other reports may be associated to this reason.

Over the past few years, frequent and extensive investigations have been conducted on the fecal genome of birds (27). The diversity of the fecal genome of birds is an important part of the gut microbiota. In this study, the wild bar-headed goose living in Tibet is an endangered protected animal. Its intestinal flora is more scientific and logical than that of the captive-bred bar-headed goose. Moreover, due to the living environment in Tibet, its intestinal flora also has unique characteristics of Tibet. Firmicutes and Bacteroidetes were the most common bacteria in all animal feces in previous studies (28), and Firmicutes are the most important microflora to promote the decomposition of cellulose by host gastrointestinal microorganisms (29). The results showed that Firmicutes and Bacteroidetes were the main bacteria encoding active enzymes of carbohydrate (30). The ratio of Firmicutes to Bacteroidetes in the gut (F/B) affects the host's ability to obtain energy from food (31). The relative abundance of Firmicutes in poultry cecum is positively correlated with body weight gain (32) and egg production performance (33). Another study showed that early use of antibiotics in chickens reduced the relative abundance of Firmicutes and increased the relative abundance of Proteobacteria. In the later stage, the immune activity of individual T cells decreases with low relative abundance of Firmicutes (32), indicating the influence of Firmicutes and Proteobacteria on the immune functions of the host. The third dominant phylum in feces is Cyanobacteria. Previously, lactic acid bacteria were added to chicken feed to improve the growth performance and immune response of chickens. The same bacteria, Cyanobacteria, Proteobacteria, *Bacteroides*, and Actinomycetes, were also detected (34). In terms of genera, the top two genera sequenced are *Bacillus* (41.5%) and *Solibacillus* (16.35%). Although *Bacillus* accounted for 41%, the study indicated no significant presence of *Bacillus*. *Bacillus* belongs to bacillus family, and the genus is *Bacillus*, which is a kind of gram-positive bacteria that can produce endophytic spores and a large amount of calcium pyridine dicarboxylic acid. The antibacterial substances produced by bacillus generally have a wide antibacterial spectrum, which can kill bacteria including drug-resistant strains, some fungi, parasites, certain viruses, and tumor cells and can bind lipopolysaccharides and neutralize endotoxins. Therefore, the researchers focused their studies on the diversity of intestinal microbes. The probiotics prepared by bacillus play an important role in the treatment of disorders in intestinal microflora, candida infection, and wound infections.

In this study, compared with the 16S sequencing results of previous reports, the focus is on the inclusion of chloroplast-containing fecal *Yanobacteria* at the phylum level. This may be due to the lack of food supply in Tibet in winter and the need to consume chloroplast-containing plants for survival. In terms of genera, *Bacillus* is the most dominant, which reduces the incidence of disease in poultry. These are the self-regulation mechanisms of bar-headed geese to adapt to life in Tibet. For wild animals raised in captivity, although wild animal resources are protected, it has caused the destruction of microbial diversity. While protecting animal germplasm resources, we should also make appropriate ecological improvements to wild animals to protect the diversity of microbial communities.

## Conclusion

In our study, we investigated the intestinal microbiome in the feces of wild bar-headed geese which provides valuable resources for further research on the gene functions of different bar-headed geese and the intestinal microbiome of wild animals. These are also valuable for genetic and high-altitude research in the Tibet Autonomous Region.

## Data Availability Statement

The datasets presented in this study can be found in online repositories. The names of the repository/repositories and accession number(s) can be found here: https://www.ncbi.nlm.nih.gov/, PRJNA777038.

## Ethics Statement

The animal study was reviewed and approved by all experimental procedures were espoused by the South China Agricultural University Animal Care Committee.

## Author Contributions

SD and SX conceived and designed the experiments and analyzed the data. JZ, RH, KM, HZ, HL, and YY provided manuscript editing. All authors statistically analyzed, discussed, critically revised the contents, and approved the final manuscript.

## Funding

This study was supported by the major science and technology projects of the Tibet autonomous region (XZ202101ZD0005N), Key R&D plan of Bayi District, Nyingchi City (2021-GX-SY-01), and the Basic Research Funds of China Agricultural University (2021TC002).

## Conflict of Interest

The authors declare that the research was conducted in the absence of any commercial or financial relationships that could be construed as a potential conflict of interest.

## Publisher's Note

All claims expressed in this article are solely those of the authors and do not necessarily represent those of their affiliated organizations, or those of the publisher, the editors and the reviewers. Any product that may be evaluated in this article, or claim that may be made by its manufacturer, is not guaranteed or endorsed by the publisher.
